# The IntegraPark Study: An Opportunity to Facilitate High-Intensity Exercise with Immersive Virtual Reality in Parkinson’s Disease Patients

**DOI:** 10.3390/jfmk9030156

**Published:** 2024-09-03

**Authors:** José M. Cancela-Carral, Pablo Campo-Prieto, Gustavo Rodríguez-Fuentes

**Affiliations:** 1 Departamento de Didácticas Especiais, Facultade de Ciencias da Educación e do Deporte, Universidade de Vigo, E-36005 Pontevedra, Spain; chemacc@uvigo.gal; 2 HealthyFit Research Group, Galicia Sur Health Research Institute (IIS Galicia Sur), SERGAS-UVIGO, E-36312 Vigo, Spain; gfuentes@uvigo.gal; 3 Departamento de Bioloxía Funcional e Ciencias da Saúde, Facultade de Fisioterapia, Universidade de Vigo, E-36005 Pontevedra, Spain

**Keywords:** exercise, Parkinson’s disease, virtual reality exposure therapy, rehabilitation, quality of life, physical therapy, mobility

## Abstract

Background: high-intensity exercise is a feasible and effective modality in the early stages of Parkinson’s disease (PD). However, there are only a few studies that have carried out this type of intervention in customizable immersive virtual reality (IVR) environments. We explore the feasibility and effects of IVR-based high-intensity training through rowing and cycling exercises on the functional capacity, quality of life, and progression of PD. Methods: a total of 12 participants (61.50 ± 10.49 years old; 41.7% female, 58.3% male; stages I–III) were part of the study, which consisted of interventions of rowing and cycling in an IVR commercial exergame (25 min; twice per week for 14 weeks). The main variables measured were functional capacity, handgrip strength, functional mobility (TUG), functional lower-limb strength (FTSST), aerobic capacity (2-min step test), quality of life (PDQ-39), and Parkinson’s disease progression (MDS-UPDRS). Results: the results showed a general improvement in handgrip strength in both hands (*p* = 0.008; d = 0.28), FTSST (*p* = 0.029; d = 0.96), and TUG times (*p* = 0.152; d = 0.22). Aerobic capacity, assessed by a 2-min step test, showed enhanced scores (*p* = 0.031; d = 0.78). Related to the PDQ-39, all dimensions of the scale were enhanced, highlighting activities of daily living (*p* = 0.047; d = 0.29) and bodily discomfort (*p* = 0.041; d = 0.37). Finally, the main symptoms of the disease were reduced, with an improvement in the parameters that show a better incidence of disease progression, such as Part IA and IB (*p* = 0.013; d = 0.29 and *p* = 0.021; d = 0.25, respectively), Part II (*p* = 0.021; d = 0.23), Part III (*p* = 0.040; d = 0.39), and Part IV (*p* = 0.013; d = 0.39). Conclusions: the therapeutic exercise (rowing and cycling), when carried out at a high intensity and in a personalized IVR scenario, appear to be a feasible and safe modality for patients with stages I–III of PD, improving their functional capacity, quality of life, and disease progression.

## 1. Introduction

Parkinson’s disease (PD) is a complex neurodegenerative disease of the central nervous system that is characterized by the appearance of motor and non-motor symptoms that progressively affect those who suffer from it [1]. It is classically considered to present five phases [2]: (1) stage 1 or initial: mild symptoms that barely affect the patient’s daily living activities (ADL); (2) stage 2: symptoms become more evident, performance of ADL worsens due to bradykinesia (slowness of movement), and the patient requires pharmacological treatment; (3) stage 3: the patient responds well to pharmacological treatment, but symptoms continue to worsen and decrease the patient’s functionality (greater difficulty with balance, more marked bradykinesia, increased falls, greater difficulty dressing, etc.); (4) stage 4: non-motor symptoms appear (especially cognitive disorders, sleep disturbances, hallucinations, etc.), medication is no longer effective, dyskinesia and motor/behavioral fluctuations appear (ON/OFF states); and (5) stage 5: standard medication is no longer responsive, severe motor and non-motor complications are present and lead to a total dependence for ADL. Currently, this five-point scale has been modified to one that is composed of seven points [3].

During the last decade, therapeutic exercise (TE) has become a non-pharmacological intervention with undeniable benefits for people with PD [4]. TE not only helps improve the body’s general physical condition, but also positively impacts several specific symptoms of the disease [5], such as (1) an improvement in mobility (exercise helps to maintain and enhance the ability to move, which is crucial for people with PD, who often experience stiffness and bradykinesia), (2) an increase in muscle strength (resistance and strength exercises can help counteract muscle weakness and improve functionality and independence in daily activities), (3) an improvement in balance and coordination (activities that challenge balance and coordination can reduce the risk of falling, a common problem for people with this condition), (4) a reduction in rigidity (regular exercise can help decrease muscle stiffness, thereby improving flexibility and range of motion), (5) cognitive benefits (some studies also suggest that exercise can have positive effects on cognitive function and mood, helping to combat depression and anxiety, which are common in this disease), (6) neuroprotective stimulation (exercise has been shown to promote the release of neurotrophic factors, which can have neuroprotective effects and improve brain health [6]), and (7) an improvement in sleep (TE can help regulate sleep patterns, improving the quality of rest [7]). To achieve these effects, it is essential that TE programs are personalized and supervised by exercise and health professionals to ensure they are safe and effective. In summary, TE is a valuable and effective tool for improving the quality of life of people with PD [8].

Recent studies on TE and PD have highlighted the importance of intensity as a fundamental factor in obtaining the aforementioned benefits [9,10,11]. It is important to remember that PD is a neurodegenerative disease, characterized as a clinical syndrome with tremors, akinesia, rigidity, and postural instability resulting from a specific underlying pathology, defined by a massive loss of pigmented dopaminergic neurons in the substantia nigra (SN) and the deposition of Lewy bodies. The presence of two of the three cardinal motor signs (tremor, rigidity, or bradykinesia) and a favorable and sustained response to L-DOPA (70–100%) are considered essential for a diagnosis of PD [12].

Based on this, personalized high-intensity exercise can help improve mobility, balance, and coordination [13], aspects that tend to deteriorate with the progression of the disease. Additionally, physical exercise routines carried out at a high intensity stimulate the release of neurotrophic factors that promote neuronal health and plasticity [6,14]. Studies have indicated that people with Parkinson’s disease who participate in intense training experience a slowing down in the progression of their motor symptoms. Improvements in their quality of life and in the ability to perform daily activities have also been observed [15]. High-Intensity Interval Training (HIIT) is a low-volume training method that consists of short periods of high-intensity exercise interspersed with rest or active recovery periods. HIIT has been shown to promote similar or greater physiological adaptations in both healthy and clinical populations, which may be appropriate for people with PD. High-intensity exercise can include activities such as cycling, rowing, running, or resistance training, and is adapted to each patient’s individual abilities. It is crucial that these programs are supervised by specialized professionals to ensure the safety and effectiveness of the training. In summary, incorporating high-intensity exercise could be an effective and transformative therapeutic strategy for people living with PD [16].

On the other hand, new technologies, such as immersive virtual reality (IVR), have been incorporated as an innovative approach to carrying out physical and cognitive therapy in various pathologies, including PD [17,18]. IVR is a technology that creates a computer-generated artificial environment that can be easily configured for therapeutic purposes [19]. This technology allows patients to focus easily on therapy without external interferences [20]. IVR exergames, which integrate games with exercise, can offer a more engaging and interactive approach to physical fitness [21,22]. The virtual reality environment allows for a wide range of virtual activities, including sports, dance, and adventure games [23]. This variety helps overcome user boredom and maintain motivation, leading to a greater adherence to physical activity. Moreover, the use of games in non-gaming contexts is a phenomenon that has been gaining attention in various areas, especially in rehabilitation settings [24]. As the technology improves, these virtual experiences become more realistic, enhancing a sense of presence, and allowing for an approximation of the specific tasks, movements, or sports modalities that have been adapted for the patient’s context [25]. Some sports modalities based on cycling [26] or rowing [27] have shown potential benefits for the health of people living with PD. However, practicing these modalities is not without risk, so their practice in controlled environments such as virtual ones could help to minimize the occurrence of accidents, in particular, falls [28], in addition to encouraging a high-intensity performance [29] that maximizes the impact of this TE on the therapeutic targets responsible for dopaminergic loss [30].

This study also has an integrative profile, fostering the autonomy and participation of those affected by this neurodegenerative disease. It aims to eliminate the barriers to exercise perceived by people with PD (e.g., the fear of falling) [31], and the barriers to the access and use of technology in the elderly population in general (i.e., the need for a prior technology mastery) [32], particularly in groups where these two disciplines could be very useful in the aging and rehabilitation processes [33].

Although there are studies that have analyzed the influence of IVR on different parameters in people with PD, such as tasks with dual-task components [22], balance and gait [34], or the work of cycling and dual-task completion [35], there are no studies that have analyzed the effects of high-intensity cycling and rowing training within an IVR environment in patients with Parkinson’s disease, which is what has been developed in the IntegraPark project. It is intended, on the one hand, to take into account the benefits from high-intensity exercise in this type of patient [16] and, on the other, its safe performance (a greater control over the high-intensity exercise to be performed with a minimal possibility of a fall occurring) and promote adherence to the treatment (previous experiences with this type of patient confirms this, as it motivates them and makes exercise more enjoyable [17,18]).

Considering this integrative approach, which combines new technological proposals and the importance of intensity in exercise therapies aimed at the group of people living with PD, the IntegraPark project aims to explore the feasibility and effects of high-intensity training based on rowing and cycling exercises on the functional capacity, quality of life, and progression of PD.

## 2. Materials and Methods

### 2.1. Study Design

This study is of a quasi-experimental design (a pre- and post-intervention study).

### 2.2. Participants

Participation was voluntary and offered to all association members who met the following inclusion criteria: are diagnosed with idiopathic PD by a neurologist; are at a disease progression stage between I and III on the Hoehn and Yahr scale; have the ability to move without assistance; and are not presenting with dementia (Montreal Cognitive Assessment -MoCA- > 26). The exclusion criteria included those who had a contraindication for physical exercise according to their physician, a severe visual or auditory disability, vertigo, or uncontrolled epilepsy. The study adhered to the ethical principles for medical research on human subjects, as outlined in the Declaration of Helsinki [36], and complied with all provisions of the Organic Law 3/2018 on Personal Data Protection and Guarantee of Digital Rights (effective 25 May 2018), ensuring the strict confidentiality of data and test results. It was approved by the Research Ethics Committee of the Galician Health Service (SERGAS), with the registration code 2023/286. All participants signed informed consent forms, and both they and their relatives or guardians were thoroughly informed about the study, its aims, and its benefits.

### 2.3. Assessment

The evaluation of the different parameters under investigation in this project was carried out twice (pre- and post-intervention assessments). Additionally, the control evaluations were conducted on the parameters indicated by the smart cycle ergometer and row ergometer (baseline, after 15 days, and final). The variables to follow were assessed.

#### 2.3.1. Sociodemographic and Pharmacological Characteristics of the Sample

An ad hoc questionnaire was conducted, collecting the following variables: age, sex, years since a Parkinson’s diagnosis, the first symptom of the disease, and any antiparkinsonian pharmacological treatment being received.

#### 2.3.2. Functional Capacity

- Handgrip strength was measured with an analogue hand dynamometer (Jamar^®^ Smart Hand Dynamometer, Patterson Medical Ltd., Nottinghamshire, UK). The participants performed this test in a standing position, holding the dynamometer in their dominant hand, and their elbows bent at 90 degrees [37].
- Functional mobility was assessed using a timed up-and-go (TUG) test [38]. This test evaluates the basic functional mobility of the subject, as well as their dynamic and static balance and their risk of falling [39].
- Functional lower-limb strength was evaluated using the five times sit-to-stand test (FTSST), which measures the time taken for an individual to stand up and sit down five times from a seated position [40].

#### 2.3.3. Aerobic Capacity

Aerobic capacity was measured with the 2-min step test. Aerobic endurance is assessed by counting the number of times the right knee reaches a height equal to the midpoint between the patella and the anterior superior iliac spine of the subject [41].

#### 2.3.4. Quality of Life

The Parkinson’s Disease Questionnaire (PDQ-39) was used to assess the participants’ quality of life [42,43]. The PDQ-39 scale assesses eight different domains: mobility (10 items), activities of daily living (6 items), emotional well-being (6 items), stigma (4 items), social support (3 items), cognitive status (4 items), communication (3 items), and bodily discomfort (3 items). The possible answers are never, occasionally, sometimes, often, or never.

#### 2.3.5. Progression of PD

The Movement Disorder Society-sponsored review of the Unified Parkinson’s Disease Rating Scale (MDS-UPDRS) was used to assess changes in symptomatology over the duration of the project [44]. It consists of four parts: Part I (non-motor experiences of daily living), Part II (motor experiences of daily living), Part III (motor exploration), and Part IV (motor complications).

### 2.4. Training Protocol

The high-intensity IVR program (IntegraPark) was conducted at the University of Vigo, specifically on the fifth floor of the Blue Building located on Benito Corbal Street in Pontevedra. The program lasted for 4 months (16 weeks), with the first and last weeks dedicated to evaluations. The frequency of the interventions were two days per week (in the morning and at least 48 h apart). In each session, the patients had to pedal/row continuously for 25 min (a 5 min warm-up, followed by 15 min for the main part, and 5 min to cool down). The participants performed a high-intensity session (HIIT mode) in an IVR environment in a sitting position. This starting position is determined by the chosen modality (rowing or cycling). This position minimizes the risk of falling. The training sessions were conducted on the first day of each week using a cycle ergometer and on the second day with a rowing ergometer with participants exerting an effort between 70 and 80% of their maximum heart rate, measured with a Mi Smart Band 4 wristband and the Zeep Life 6.9.5 version app, and with a perceived exertion rate of 8–9/10 on a modified Borg scale [45].

**Figure 1:**
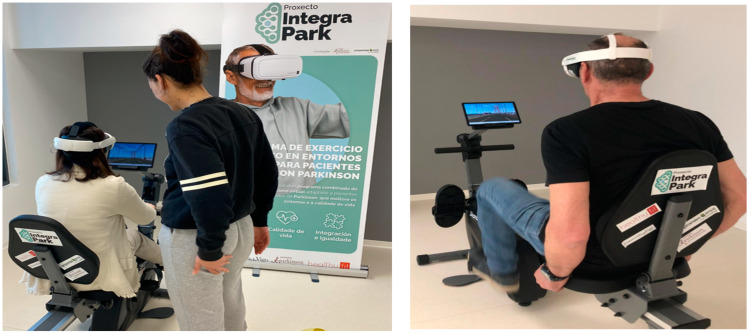
The participants performing two exercise modalities (rowing and cycling) in immersive virtual reality environments with a member of the research group supervising the session.

**Figure 2:**
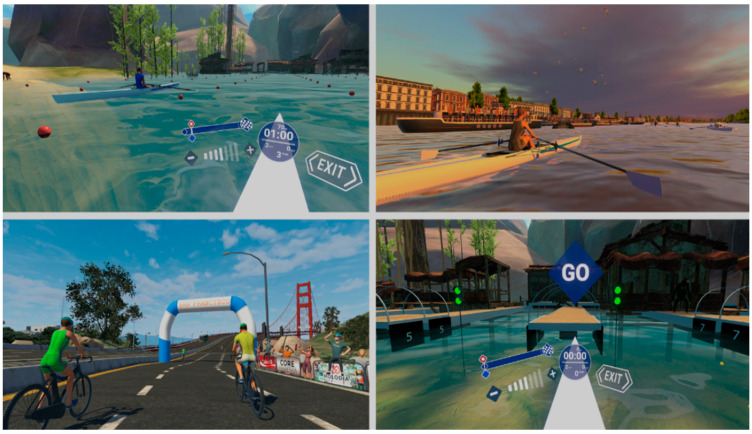
Screenshots of the different exergame modalities used in the intervention (IntegraPark program).

To carry out the IVR program, a head-mounted display (Meta Quest III, Oculus VR, Menlo Park, CA, USA) and HoloFit software 4.7.0.8 version (available in the library at www.meta.com, accessed on 1 March 2024) were used. When synchronized with the smart cycle/row ergometer (Vinur Plus III, Skandika, Essen, Germany), this allowed the patient to pedal and row through virtual environments, such as different European cities (London, Paris, Venice, etc.), natural landscapes, or sports competitions, while simultaneously performing small cognitive tasks. They performed the activities in the HIIT mode of the exergame. Following the manufacturer’s instructions, a play area of approximately 5 m^2^ was defined, and the therapist could see the session with a tablet (see Figure 1 and Figure 2).

All the participants continued with their regular activities at the patients’ association throughout the duration of the program.

### 2.5. Data Analysis

A descriptive analysis of the sample characteristics was conducted using measures of central tendency (mean), dispersion (standard deviation), and percentage. The Shapiro–Wilk test was used to determine whether the data were normally distributed (*p* > 0.05). Therefore, based on the data distribution, a *t*-test (for normally distributed data) was used to find statistical differences in the calculated parameters between pre-intervention and post-intervention evaluations. All analyses were performed globally. The effect size was calculated using Cohen’s statistic. Cohen classified the effect sizes as small (d = 0.2), medium (d = 0.5), and large (d ≥ 0.8). The data obtained were processed with IBM-SPSS statistical software v.25. The value of statistical significance was set at *p* < 0.05.

## 3. Results

A total of 12 participants (61.50 ± 10.49 years old; 41.7% female, 58.3% male) took part in the study. All of them belonged to the provincial Parkinson’s Association of Pontevedra (APRO-PARK), and 58.3% were at stage III of the disease (see Table 1).

The intervention program was completed by all 12 patients who participated in it, with 100% of the scheduled sessions performed (100% adherence). None presented any adverse symptoms to the VR exposure or to the high-intensity exercises.

Table 2 shows the values recorded at the two evaluation moments (pre- and post-intervention) for the functional parameters (handgrip strength, functional lower-limb strength, functional mobility, and aerobic capacity), quality of life (8 dimensions: mobility, activities of daily living, emotional well-being, stigma, social support, cognition, communication, bodily discomfort), and progression of PD (5 parts: Part IA: non-motor aspects of experiences of daily living, Part IB: non-motor aspects of experiences of daily living, Part II: motor aspects of experiences of daily living, Part III: motor examination, Part IV: motor complications).

The results obtained for functional capacity reflect an increase in the handgrip strength in both hands, with a greater increase in the dominant hand. Regarding functional lower-limb strength, the data also shows an improvement of 24.57% in this parameter (FTSST). Functional mobility/dynamic balance was evaluated using a TUG test. The results obtained from this test indicate that the participants demonstrated an improvement in this parameter of 2.21%. The patients’ aerobic capacity was evaluated with the 2-min step test. The values obtained at both evaluation points reflect that, after 14 weeks of intervention, the patients registered an improvement of 9.76%.

Table 2 also shows the results obtained in the patients’ quality of life parameters. This parameter was evaluated with the PDQ-39 scale. The results show an improvement across all dimensions of the scale, with the dimensions of mobility (39.96%), emotional well-being (43.88%), and cognition (48.07%) standing out. Regarding the effect of the exercise program on the disease’s progression, it is worth noting that the parameters showing the greatest improvement were Part IA: non-motor aspects of experiences of daily living (30.87%) and Part IV: motor complications (25.09%).

Table 3 shows the inferential analysis between the two evaluation moments, with those variables reflecting significant differences and their degree highlighted. Based on this analysis, it can be shown that all variables defining functional capacity show significant improvements (*p* < 0.05), except for the TUG test for dynamic balance/functional mobility (*p* = 0.152). Regarding quality of life, it should be noted that out of the eight dimensions of PDQ-39, only two show significant improvement, which are activities of daily living (*p* = 0.047) and bodily discomfort (*p* = 0.041). The inferential analysis of disease progression reflects that all parts of the MDS-UPDRS show significant improvement, with Part IA: non-motor aspects of experiences of daily living (*p* = 0.013) and Part IV: motor complications (*p* = 0.013), in particular, standing out.

## 4. Discussion

The IntegraPark program aimed to explore the feasibility and effects of high-intensity training (rowing and cycling) performed in IVR environments on the functional capacity, quality of life, and progression of PD. Participants with PD adhered to the prescribed exercise intensity (70–80% of their maximum heart rate/perceived exertion rate of 8–9/10 on a modified Borg scale) for the program, which lasted 14 weeks at a frequency of two days per week. The results show the feasibility of using IVR for carrying out high-intensity exercise programs in patients with PD at stages I to III. The use of such a program was safe and had generated an improvement in the functional capacity and quality of life of the participants, and may have reduced adverse symptoms and the progression of the disease.

Previous studies [14,29,46,47] have highlighted the beneficial effects of high-intensity training in patients with PD, such as improving peak oxygen consumption, lower limb strength, or motor function. However, as far as we know, none of them has used rowing as a physical exercise therapy and only one has used IVR technology for performing the therapy [29]. The use of this novelty tool allowed for early-stage Parkinson’s patients to perform a physical therapy, based on a combination of rowing and cycling exercises, in complete safety, generating a high adherence to the program. IVR has allowed us to customize the exercise practice environment (nature, city) according to the preferences and interests of the patient while maintaining stable work guidelines for all of them. Other studies conducted in real environments [14,48] have demonstrated the influence of high-intensity programs on the aerobic capacity of the population diagnosed with PD, but so far, no high-intensity physical exercise program carried out in an immersive virtual environment has evaluated its influence on aerobic capacity. In our case, the IntegraPark program, a combination of high-intensity rowing and cycling exercises, generated a significant improvement in aerobic capacity (*p* = 0.031). Other functional parameters that improved during the IntegraPark program include handgrip strength and lower-body strength. The level of lower-body strength has also been evaluated in other research studies [15,16,48] following the use of a high-intensity physical exercise program. Improvements were observed in two of them [15,16], for example, in aerobic capacity measured by a 6-min walking test or cycle endurance, respectively, which are closely related to the strength and functionality of the lower limbs. However, the study by Demonceau et al. [48] did not report any improvements in the strength of knee extensors or flexors after six sessions of HIIT on a cycle ergometer. In addition, handgrip strength was evaluated using the protocol of Bjerregaard et al. [37]. The results in our study showed significant improvement in both the dominant and non-dominant hands. However, Kim et al. [49] conducted a high-intensity physical exercise program in a Parkinson’s population and did not observe significant differences in the handgrip parameters. This difference in behavior between the two studies may be due to the fact that their programs were mainly oriented toward the lower body [49], whereas in the IntegraPark program, the rowing component involved gripping the bar to perform the exercise, causing an isometric contraction of the muscles throughout the execution of the exercise.

Both issues, handgrip and lower body strength, are relevant in patients with PD. It must be remembered that Wang and Chen [50], although focused on community-dwelling older adults, point out that handgrip strength deficiency is linked to disability of a lesser or greater degree. For their part, Skinner et al. [51] reinforce the importance of strength in the lower limbs in order to have good motor control and, therefore, effective ambulation.

Functional mobility/dynamic balance was evaluated using a TUG test [38,39]. The IntegraPark program generated improvement; however, this is not significant, as reported in the study by Yun et al. with IVR [22]. In contrast, Feng et al. [34] achieved significant results with both the TUG test and the Berg Balance Scale and the Functional Gait Assessment, although it seems that VR, not IVR, was used in the experimental group. Similar results were obtained in other studies that developed high-intensity physical exercise programs [15,48]. However, another study [52] showed improvement with a TUG test, with a reduction of 0.54 s (15.59%) in the time required, although they reported no significant improvement (+10.81%) in the Activities-specific Balance Confidence Scale.

It is possible that the sample size and the load and type of work applied in the program were not sufficient to generate significant improvement, an aspect that should be studied in the future. In a previous study of high-intensity forced cycling and IVR [29], improvements were obtained that were considered the minimum detectable change to be considered a true change.

Quality of life is also an important parameter in determining the impact of the pathology of PD on patients. A number of investigations have evaluated this parameter following the performance of high-intensity physical exercise programs and have observed no differences in patients’ quality of life [14,48]. In the case of the IntegraPark program, the results obtained by the previously mentioned researchers are confirmed. However, if we delve into the dimensions of the PDQ-39, we can observe that some items, such as activities of daily living and bodily discomfort, show significant improvement differences. This aspect requires further study in future research, perhaps exploring possible correlations with the typology of the sample.

Finally, the MDS-UPDRS is a 4-subscale, a combined scale that comprehensively assesses the symptoms of PD and provides and overview of the progression and impact of the disease. It consists of the following four parts: Part I, non-motor experiences of daily living, including thirteen items (six semi-structured interview items and seven self-reported items); Part II, motor experiences of daily living, including thirteen self-reported items; Part III, motor examination, including eighteen items (thirty-three scores); and Part IV, motor complications, including six items assessed in a semi-structured interview [44]. The performance of the IntegraPark program has generated significant differences in all four parts of the progression of the disease scale. This scale is specific to Parkinson’s disease, and is widely used by researchers. For example, Duplea [53] found improvements in both the HIIT and continuous moderate-intensity training groups (12.8 and 8.2 points, respectively), but no significant differences between the groups. Marusiak et al. [54] reported improvement in the bradykinesia subsection after a HIIT program compared to the usual care (*p* < 0.001). The same study also showed improvement in the HIIT group in Part II of the UPDRS (motor aspects of daily living), although no differences were found between the groups, nor were there any changes in the activities of daily living scale. However, Uygur et al. [52] reported a 20.14% improvement (3.5 points) in Part III of the UPDRS and a 15.1% improvement in the bradykinesia subsection of the UPDRS. Furthermore, Feng et al. [34] and Chang et al. [35] also found significant results in Part III of the UPDRS (which is the only one they used).

In view of our results and the existing evidence in the literature, high-intensity exercise programs seem to have a considerable impact on disease progression measured with the MDS-UPDRS and, in line with what has been found in recent articles [29,55], it seems to be able to consolidate itself as a valid complement to specialized physiotherapy for PD.

However, high-intensity cycling work is not protocolized for people with PD. For example, there are studies where only three sessions of 40 min were applied at 50–80% of the reserve heart rate [56]. Others only applied one session of 40 min to 60–70% of reserve heart rate [57], or proposed four sessions/week of 60 min for four weeks of high intensity, adjusted to the anaerobic threshold of each patient, where work was conducted in five intensity zones between 60–70% and 100–110% of said threshold [58]. It is possible this is one of the future lines of research in this field.

On the other hand, the chosen sport modalities have also been shown to influence positive results, especially in cycling, for which there is strong evidence of its successful use [59,60]. Regarding rowing, the evidence is scarce, undoubtedly at a disadvantage to the pedaling modality aimed at crucial aspects of PD, such as gait, balance, and the risk of falling.

Furthermore, it should be noted that the portable IVR device Meta Quest 3, when applied as a facilitator of exercise and addressed to PD [61], has been reliable and well-tolerated by the participants, and is in-line with the evidence found by this research group’s use of the previous model (Meta Quest 2).

### Limitations

These results are promising, but this study presents some limitations. First, since it is a pilot study, the sample is too small and is not representative of the population with PD (those at stages I–III and in good general condition). In addition, as the intervention was only two sessions per week, maybe it would be necessary to include more sessions to explore the safety and feasibility of the program. Another limitation is the absence of a control group to make comparisons between groups of the evaluated variables and the type of exercise proposed (cycling and rowing). Finally, a follow-up assessment could determine the possible longevity of the effects generated by the IntegraPark program.

## 5. Conclusions

The TE proposed (rowing and cycling for patients with PD), carried out at a high intensity (between 70 and 80% of their maximum heart rate) and in a personalized immersive virtual environment, for 14 weeks at a frequency of twice per week, supervised by a professional, appears to be a feasible and safe program for patients with PD in stages I–III of the disease. The strengths of this study are that the types of exercise and the intensity employed improved the functional capacity (muscle strength, balance, and functional mobility), aerobic capacity, and some dimensions of quality of life, as well as the symptoms and progression of the disease in patients with PD. In addition, this program has also allowed people at risk for exclusion to practice sports modalities in a safe and fun way, increasing their self-esteem and participation. Future quality studies are needed to investigate the effects of different HIIT protocols on other physiological and clinical parameters (stress, anxiety, cognitive decline, or its use in the advanced stages of the disease), and to explore further safe methods to facilitate access and long-term adherence for these patients.

## Figures and Tables

**Table 1:** The demographic and clinical characteristics of the participants.

		Mean ± SD/%
Age (years)		61.50 ± 10.49
Sex	Male	58.3%
	Female	41.7%
BMI (kg/m^2^)		27.86 ± 4.09
Academic Level	Higher education	41.7%
	Vocational training	16.6%
	University	41.7%
Personal History of Parkinson’s	None	25.0%
	Direct family member	33.3%
	Other family	25.0%
	I do not know	16.7%
Years diagnosed with Parkinson’s	≤5.00	66.6%
	>6.00	33.3%
H&Y stage	Stage I	25.0%
	Stage II	16.7%
	Stage III	58.3%
First symptom	Tremors (%)	41.6%
	Rigidity (%)	25.0%
	Bradykinesia (%)	16.6%
	Other (%)	16.6%
Pharmacology	LEDD (mg)	634.21 ± 301.65

BMI: body mass index; H&Y: Hoehn and Yahr; LEDD: levodopa equivalent daily dose; SD: standard deviation.

**Table 2:** The Main results in the variables evaluated (functional capacity, aerobic capacity, quality of life, and disease progression) at pre- and post-intervention moments.

			Pre-Intervention				Post-Intervention			
		%	Mean	SD	Min.	Max.	Mean	SD	Min.	Max.
**Functional capacity**										
Dominant hand	Right	83.3%								
	Left	16.7%								
Handgrip [dominant hand] (kg)			36.17	11.82	20.00	48.00	39.22	10.15	20.00	49.22
Handgrip [non-dominant hand] (kg)			36.08	11.27	14.00	47.00	38.80	9.61	22.08	48.00
Five times sit-to-stand (s)			7.65	2.53	3.92	12.00	5.77	1.36	3.94	7.73
Timed up-and-go (s)			6.32	0.67	5.28	7.22	6.18	0.56	5.28	7.17
**Aerobic capacity**										
2-min step test (*n*)			93.44	11.07	81.00	110.00	102.56	12.30	84.00	126.00
**Quality of Life (PDQ-39)**										
Mobility (10, #1–10)			4.58	6.97	0.00	17.50	2.75	4.26	0.00	10.50
Activities of daily living (6, #11–16)			6.94	11.08	0.00	29.17	4.22	7.38	0.00	19.13
Emotional well-being (6, #17–22)			2.78	4.30	0.00	8.33	1.56	2.45	0.00	5.33
Stigma (4, #23–26)			2.08	5.10	0.00	12.50	1.68	4.12	0.00	10.10
Social support (3, #27–29)			1.39	3.40	0.00	8.33	1.22	2.99	0.00	7.33
Cognition (4, #30–33)			1.04	2.55	0.00	6.25	0.54	1.33	0.00	3.25
Communication (3, #34–36)			5.28	12.93	0.00	31.67	4.61	11.28	0.00	27.64
Bodily discomfort (3, #37–39)			12.50	13.69	0.00	33.33	8.27	9.17	0.00	23.15
Total			4.57	6.55	0.31	17.60	3.10	4.88	0.12	12.89
**Disease progression (MDS-UPDRS)**										
Part IA: non-motor aspects of experiences of daily living			2.17	2.40	1.00	7.00	1.50	2.26	0.00	6.00
Part IB: non-motor aspects of experiences of daily living			3.67	3.44	1.00	8.00	2.83	3.37	0.00	8.00
Part II: motor aspects of experiences of daily living			3.33	3.88	1.00	11.00	2.50	3.27	0.00	9.00
Part III: motor examination			6.67	4.76	1.00	13.00	5.00	3.69	1.00	10.00
Part IV: motor complications			2.67	1.75	1.00	6.00	2.00	1.67	0.00	5.00

**kg**: kilograms; **max.**: maximum; **MDS-UPDRS**: MDS-Unified Parkinson’s Disease Rating Scale; **min.**: minimum; ***n***: number of repetitions; **PDQ-39**: Parkinson’s Disease Questionnaire; **s**: seconds; **SD**: standard deviation.

**Table 3:** An inferential analysis of the effects of the IntegraPark program on the variables evaluated (functional capacity, aerobic capacity, quality of life, and disease progression) at the pre- and post-intervention moments.

	Paired Differences					Cohen’s d
			95% CI			
	Mean	SD	Lower	Upper	Sig.	
**Functional capacity**						
Handgrip [dominant hand] [(kg)]	2.86667	3.50784	5.09544	0.63789	0.008	0.28
Handgrip [non-dominant hand] [(kg)]	1.21167	1.82738	2.37273	0.05061	0.021	0.26
Five times sit-to-stand [(s)]	−1.32333	1.79348	0.05526	−2.70193	0.029	0.96
Timed up-and-go [(s)]	−0.13778	0.37586	0.15113	−0.42669	0.152	0.22
**Aerobic capacity**						
2-min step test [(*n*)]	9.11111	12.66338	18.84504	0.62282	0.031	0.78
**Quality of Life (PDQ-39)**						
Mobility (10, #1–10)	1.83333	2.73252	−1.03427	4.70094	0.081	0.33
Activities of daily living (6, #11–16)	2.72778	3.72735	−1.18383	6.63939	0.047	0.29
Emotional well-being (6, #17–22)	1.22278	1.94045	−0.81360	3.25915	0.092	0.36
Stigma (4, #23–26)	0.40000	0.97980	−0.62823	1.42823	0.182	0.08
Social support (3, #27–29)	0.16722	0.40961	−0.26264	0.59708	0.182	0.05
Cognition (4, #30–33)	0.50000	1.22474	−0.78529	1.78529	0.182	0.25
Communication (3, #34–36)	0.67167	1.64524	−1.05491	2.39824	0.182	0.05
Bodily discomfort (3, #37–39)	4.22833	4.77188	−0.77945	9.23612	0.041	0.37
Total	4.57	6.55	−1.03427	4.70094	0.081	0.25
**Disease progression (MDS-UPDRS)**						
Part IA: non-motor aspects of experiences of daily living	0.66667	0.51640	0.12474	1.20859	0.013	0.29
Part IB: non-motor aspects of experiences of daily living	0.83333	0.75277	0.04335	1.62332	0.021	0.25
Part II: motor aspects of experiences of daily living	0.83333	0.75277	0.04335	1.62332	0.021	0.23
Part III: motor examination	1.66667	1.86190	−0.28728	3.62061	0.040	0.39
Part IV: motor complications	0.66667	0.51640	0.12474	1.20859	0.013	0.39

**CI**: confidence interval; **kg**: kilograms; **MDS-UPDRS**: MDS-Unified Parkinson’s Disease Rating Scale; ***n***: number of repetitions; **PDQ-39**: Parkinson’s Disease Questionnaire; **s**: seconds; **SD**: standard deviation.

## Data Availability

The raw data supporting the conclusions of this article will be made available by the authors on request.
